# The choice of first-line Chronic Myelogenous Leukemia treatment

**DOI:** 10.1007/s00277-015-2321-3

**Published:** 2015-03-27

**Authors:** Carmen Fava, Giovanna Rege-Cambrin, Giuseppe Saglio

**Affiliations:** Division of Hematology and Internal Medicine, Department of Clinical and Biological Sciences, University of Turin, “San Luigi Gonzaga” University Hospital, 10043 Orbassano, Turin Italy

**Keywords:** CML, BCR-ABL, Ph-chromosome, TK inhibitors

## Abstract

Imatinib has represented a revolution in the treatment of chronic myeloid leukemia (CML), inducing an overall survival never seen with previous therapies. However, with the commonly used dosage of 400 mg, one third of the treated patients does not reach the criteria associated with an optimal outcome and could potentially benefit from a different treatment strategy. Several trials exploring modified imatinib-based treatments or second-generation tyrosine-kinase as front-line therapy have been performed. In some studies, high-dose (800 mg per day) or dose-adapted imatinib or imatinib plus interferon was reported to be able to induce better cytogenetic and molecular responses compared with standard-dose imatinib, although no improvements in progression-free survival (PFS) or overall survival (OS) have been so far reported. At the moment, these approaches are still considered investigational. On the other side, on the basis of their capacity to induce very fast and deep molecular responses, including major molecular responses (MMRs) and the newly defined very deep molecular responses MR^4^ and MR^4.5^, and to prevent at least part of the early progressions to AP/BC that still occur during the first 2–3 years from diagnosis, dasatinib and nilotinib have been approved and registered by FDA and EMA as the first-line therapy for CML patients, opening the possibility to use different therapeutic strategies for newly diagnosed CML patients and a consequent intense debate among hematologists.

## Introduction

The impressive rates of complete cytogenetic responses (CCyRs) achieved the consequent long-term overall survival (OS) observed in the treated patients, and the good tolerability led imatinib, the first tyrosine-kinase inhibitor (TKI) used for the therapy of chronic myeloid leukemia (CML), to become the standard of care and the most widely used frontline therapy for CML patients in chronic phase at the dosage of 400 mg per day [1, 2]. The most relevant data of the 8-year follow-up of the IRIS study that have also been confirmed by other studies and by independent retrospective analysis performed on patients outside clinical trials show a cumulative CCyR rate of 83 % and an estimated OS rate of 85 %, which is far better from what was observed before the introduction of this drug [3–6]. This result may be ascribed to a substantial decrease in the number of the progressions to accelerated phase or blast crisis observed in the patients treated with imatinib. All records indeed suggest that progressions to a more advanced phase of the disease still represent the major cause of death for CML patients, being still incurable in most cases even in the tyrosine-kinase inhibitor (TKI) era [7]. With imatinib therapy, the occurrence of progression drops from an expected rate of approximately 15 % per year to a rate of 2–3 % per year, and only for the first 2–3 years of treatment as during the subsequent years, events of progression are really occasional [3]. This is certainly due to the great reduction of the leukemic mass observed in most of the imatinib-treated patient that in some few cases can also result in an apparent disappearance of the leukemic clone, but also to the fact that imatinib, inhibiting the BCR-ABL tyrosine kinase (TK) activity that plays a major role in determining the genomic instability of the leukemic cells, may per se be able to slow the propensity to progress [8].

It has been demonstrated that the patients who better benefit from the TKI therapy with imatinib are those who achieve and maintain CCyR for at least 2 years, as in these cases, the OS is similar to that of a control population without leukemia [9]. On the other side, various analyses have shown that patients who do not achieve good cytogenetic or molecular responses to imatinib at defined time points have a worse outcome, characterized by an increased risk of relapse, of progression and of death [10, 11]. Based on these principles, a panel of CML experts on behalf of the European Leukemia Net (ELN) as well as members of the National Comprehensive Cancer Network (NCCN) have previously established and more recently revised treatment milestones to be achieved during CML treatment with TKIs [12, 13]. This obviously implies that, to optimize CML treatment with TKIs, an appropriate and timely follow-up with cytogenetic and standardized molecular methods of adequate reliability is needed [14–16]. In particular, molecular monitoring of BCR-ABL transcript levels by real-time quantitative PCR (RQ PCR) is progressively becoming the most useful and precise way to monitor CML patients. With respect to conventional cytogenetic analysis, RQ PCR can not only allow to monitor the first steps of reduction of the leukemic burden occurring within the first months of TKI therapy, but it may also allow to estimate the amount of the residual disease once CCyR is achieved, as the sensitivity that can be reached with the present RQ PCR procedures in a sample of good quality is in most cases between 1 × 10^−4^/10^−5^ that corresponds to an amount between 2 and 3 logs below the threshold of the achievement of CCyR [14]. According to the established international scale (IS), the relevant BCR-ABL% to be achieved are 1 % (2-log reduction with respect to the median BCR-ABL amount present at diagnosis and that roughly corresponds to the threshold of CCyR), 0.10 % BCR-ABL (major molecular response (MMR)), and 0.01 % and to 0.0032 % BCR-ABL corresponding, respectively, to MR^4^ (4-log reduction) and MR^4.5^ (4.5-log reduction) [14–16].

Probably, even in our days, the attainment of CCyR or 1 % BCR-ABL can still be considered the most significant response to target, as this goal has been demonstrated to be associated to the highest probability of long-term survival for CML patients [17–19]. On the other side, several sets of data did not appear to support the notion that deeper responses, as the achievement of level of BCR-ABL^IS^ ≤0.1 % (MMR) may indeed improve OS relative to achieve CCyR without MMR [17, 18]. More recently, however, a 4-year landmark analysis performed within the context of the German CML-study IV suggests that the patients who after 4 years were able to achieve a stable MR^4.5^ molecular response, at 8 years, show a statistically significant better survival with respect to those patients who have simply achieved CCyR, but not MMR [19]. If these results will be confirmed, MR^4.5^ will represent a new molecular predictor of long-term outcome. In any case, it has been clearly established by several clinical studies that a stable deep molecular response (at least MR^4^ or even better MR^4.5^) is requested to obtain a long-lasting treatment-free remission (TFR) that is progressively becoming the new treatment goal for CML patients [20, 21]. Thus, the achievements of MMR and of MR^4.5^ in addition to CCyR and MMR are appealing targets to pursuit, as they predict for more durable and stable responses and can also open the possibility to try to stop the therapy.

It is noteworthy that many studies, particularly in more recent years, have indicated that early cytogenetic and molecular responses within the first year of therapy represent the strongest prognostic parameters [18, 22–24], not only in terms of OS, progression-free survival (PFS), or event free survival (EFS) but also in terms of possibility of achieving deeper molecular responses and therefore the possibility of discontinuing treatment without molecular relapse(TFR) [20]. Based on these observations, the last editions of the ELN and NCCN recommendations have modified with respect to the past the time points at which the expected response goals should be met to match the criteria for optimal response [12, 13]. Whereas, previously, only hematologic remission and some degree of cytogenetic response were expected after 3 months of TKI therapy, partial cytogenetic response (PCyR) after 6 months and CCyR after 1 year, in the last editions of both ELN and NCCN recommendations, to be considered “optimal responders”, the patients should at least be in partial cytogenetic response (PCyR) and/or below the roughly corresponding 10%^IS^ BCR-ABL threshold after 3 months of therapy, at least in CCyR and/or below the 1%^IS^ BCR-ABL level after 6 months of therapy and at least in MMR after 1 year of therapy and thereafter show a continuous decline of the BCR-ABL level until the achievement of deeper responses like MR^4^ or MR^4,5^ [12, 13]. Indeed, many studies suggest that the most clinically relevant target to be achieved during TKI therapy is represented by a reduction of the BCR-ABL transcript level below 10%^IS^ at 3 months, as this is associated with a high statistically significant difference in terms of OS and PFS [18, 22–24].

Even simply based on this parameter, it appears that approximately one third of CML patients do not show an optimal response to imatinib therapy and they are therefore facing a statistically significantly higher risk of an inferior outcome in terms of EFS, PFS, and also OS (approximately 80 % at 5 years with respect to >95 % of those below 10 % BCR-ABL at 3 months) [18, 22–24]. Actually, it is true that most of these patients (approximately 80 %) will only show a delayed response and that they will simply require a switch to treatment with a second-generation TKI to achieve an optimal response in approximately 40–50 % of the cases [25, 26]. However, it should also be considered that approximately 15–20 % of them in a short time will progress to a more advanced phase of the disease and will die [18, 22–24]. In any case, several reports including IRIS have shown that after 8 years from diagnosis, only approximately 55–60 % of the patients who started with imatinib are still on treatment with this drug [3, 5]. In addition to the cases of failure, of progression, and of death, the reasons for discontinuation include also 10–12 % of patients who show adverse events (AEs) and are intolerant to imatinib treatment and should be moved to the treatment with another TKI [3].

In is also noteworthy that the percentage of the patients who do not respond optimally to imatinib may vary according to the initial clinical and hematological features that determine their initial risk category, as established by Sokal’s, and Euro and also by the more recent EUTOS score [27–29]. In the IRIS study, patients with low-, intermediate-, or high-risk Sokal’s score showed significantly different response rates as 5-year CCyR (89, 82, and 69 %, respectively: *P* < 0.001) and progression to advanced disease (3, 8, and 17 %, respectively: *P* = 0.002) [1].

Based on all these considerations, several clinical trials aiming to improve the first-line treatment of patients with chronic phase CML have been performed or are at present ongoing. The therapeutic strategies that are tested include the first-line administration of the second-generation TKIs originally used as second-line therapy or modified imatinib-based regimens, as higher dosages of imatinib from the start or combinations of imatinib with other drugs, namely, interferon-alpha (IFN-α). At present, only the use of the second-generation TKIs nilotinib at the dosage of 300 mg BID and of dasatinib 100 mg OD have been approved and registered as the first-line therapy in several countries and are also included in the ELN and NCCN recommendations, whereas the other two quoted options still remain investigational [12]. As patients with CP CML are now having a very long survival and very long follow-ups are consequently required before the efficacy of these alternative treatment options could be measured in terms of OS, important surrogate markers as the rates of CCyR, MMR, MR^4^, and MR^4.5^ achieved at relevant time-points, the more recent parameters of early molecular response (EMR) as well as the more traditional event free survival (EFS) and progression-free survival (PFS) parameters have been frequently used as way to evaluate the relative responses and to compare results. However, in order to get a correct information, it is important to consider that the methods to asses and to report the rate of responses can sometimes vary and that the definitions of the EFS and PFS may change substantially according to the protocol in different trials and may therefore introduce bias difficult to perceive in the comparative evaluation of the results [30, 31]. Considering this potential limitation, we will now review the main treatment options to imatinib 400 mg OD as the first-line therapy for CP CML patients currently available or explored in clinical trials.

## Second-generation TKIs in first-line treatment

Following the success of imatinib, three different second-generation BCR-ABL inhibitors, more potent than imatinib, have been tested as the first-line therapy to try to overcome the residual resistance still shown by some patients to imatinib and to further improve the outcome of CP-CML patients [32]. These drugs were TKIs already approved as second-line therapy for imatinib-intolerant or imatinib-resistant patients, namely, dasatinib (Sprycel, Bristol-Myers Squibb) [33], a dual BCR-ABL and SRC inhibitor, nilotinib (Tasigna, Novartis) [34], a potent and more selective BCR-ABL inhibitor and bosutinib (Bosulif, Pfizer) another potent dual BCR-ABL and SRC inhibitor [35].

All these drugs when used as second-line therapy showed a distinct, but substantially good toxicity profile and were able to induce a CCyR rate of 40–50 % in patients with primary or secondary resistance to imatinib [25, 26], also when this was due to the presence of clones with most of the BCR-ABL mutations able to confer resistance to imatinib, with some notable exceptions like the T315I mutation [36].

The efficacy and the toxicity of nilotinib and dasatinib as the first-line therapy were initially assessed in phase 2 studies that have now reached a rather long follow-up [37–39]. The results obtained in 73 newly-diagnosed CP-CML patients treated with nilotinib 400 mg twice a day by the GIMEMA CML working party showed CCyR achievement at 3 months in 78 % of the patients and in 96 % at 6 months, whereas the MMR rates observed were 52 and 66 %, respectively, at the same time points and 85 % at 12 months [37]. Similarly, results of 100 newly diagnosed CML patients treated at the MD Anderson Cancer Center with nilotinib 400 mg twice daily (BID) showed, with a median follow-up 29 months (range 1–73), a cumulative CCyR rate of 93 % a rate of MMR of 73 % and a CMR rate (defined according to the previous ELN criteria as undetectable hybrid transcripts with a sensitivity of at least 10^−4/−5^) of 33 % [37]. At the same institution, 86 newly-diagnosed patients were treated with dasatinib 50 mg twice daily (BID) or 100 mg QD [39]. With a median follow-up of 24 months, most patients achieved a rapid CCyR (94 % at 6 months), with a cumulative CCyR ratio of 98 %. After 12 and 18 months, MMR was achieved by 71 and 79 % of patients [39]. The toxicity profile with dasatinib was also favorable, with a better tolerability with dasatinib QD vs BID dosing.

ENESTnd is a phase 3, randomized, open-label, multicenter study comparing the efficacy and safety of nilotinib with imatinib in patients with newly diagnosed CML that has now reached the fifth year of follow-up [40, 41]. The trial included 846 patients randomly assigned 1:1:1 to nilotinib 300 mg BID (*n* = 282), nilotinib 400 mg BID (*n* = 281), or imatinib 400 mg/day (*n* = 283). MMR at 12 months was the primary endpoint. Patients were also stratified by Sokal’s risk score, which resulted in equal distributions of low-, intermediate-, and high-risk Sokal’s scores in each arm of the trial. Efficacy results were presented in the intent-to-treat (ITT) population. The MMR rate at 12 months was significantly higher for nilotinib 300 mg BID (44 %, *P* < 0.0001) and nilotinib 400 mg BID (43 %, *P* < 0.0001) than for imatinib (22 %). As this was the primary endpoint of the study, nilotinib 300 mg BID was approved by FDA and EMA and it is now registered as the first-line therapy in several countries. Responses were rapidly achieved with nilotinib, with 6-month MMR rates of 33, 30, and 12 % for nilotinib 300 mg BID, nilotinib 400 mg BID, and imatinib, respectively. These higher responses were also associated with significantly fewer progressions to AP/BC with nilotinib than with imatinib as already observed during the first year of the study [39]. After a minimum follow-up of 5 years, rates of MMR and MR^4.5^ continue to be significantly higher in both nilotinib arms versus the imatinib arm (MMR 77 and 77.2 versus 60 % and MR^4.5^ 53.5 and 52.3 versus 31.4 %), with more than half of the nilotinib-treated patients achieving MR^4.5^ by 5 years [40]. When considering progression events occurring during treatment and after treatment discontinuation, rates of freedom from progression to AP/BC remain statistically higher in the nilotinib-treated patients (96.3 and 97.8 % for nilotinib versus 92.1 % imatinib). However, although estimated rates of OS are higher in the nilotinib arms versus the imatinib arm (93.7 % nilotinib 300 mg BID, 96.2 % nilotinib 400 mg BID, and 91.7%imatinib), at the moment, they do not reach a statistically significant difference. The frequency of adverse events (AEs) leading to discontinuation was lowest in the nilotinib 300 mg BID arm (12.2 %), followed by the imatinib arm (13.9 %) and the nilotinib 400 mg BID arm (19.9 %) [40]. However, the occurrence of cardiovascular events, which have been frequently reported in association with nilotinib therapy, has been more frequently observed in both nilotinib arms that in the imatinib arm, although these events (including all definitions of different gravity and also cerebrovascular events and PAD, peripheral arterial disease) are definitely more frequent in the 400 mg BID arm than in the 300 mg BID arm (7.5 % in the nilotinib 300 mg BID, 13.4 % nilotinib 400 mg BID versus 91.7 in the imatinib arm) [40]. In conclusion, the 5-year follow-up data confirm the sustained efficacy of frontline nilotinib over imatinib as front-line therapy including achievement of earlier and deeper molecular responses and increased freedom from progression to AP/BC. These results can be particularly relevant also in light of the reported option for some patients attaining a very low level of residual disease (MR^4.5^ or lower) to discontinue the therapy without recurrence of the disease at least for a relevant period of time [20]. It is also relevant that, comparing only nilotinib 300 mg BID and imatinib 400 mg OD at 3 months, 91 % of patients in the nilotinib arm versus 67 % in the imatinib arm achieved BCR-ABL transcript levels ≤10 and 56 % versus only 16 % of patients achieved already BCR-ABL transcript levels ≤1 % [23]. The initial molecular response correlates also with progression to AP/BC and with OS in both treatment arms, as among the patients who achieved ≤10 % BCR-ABL at 3 months, only 3 progressed on treatment whereas 9 of 111 patients who achieved >10 % at 3 months progressed. These results clearly show the relevance to evaluate early molecular response at 3 months [23].

Dasision is a phase 3, randomized, open-label, multicenter study comparing the efficacy and safety of dasatinib 100 mg OD as the first-line therapy with respect to that of imatinib [42]. Even this study has now achieved a minimum follow-up of 5 years [43]. Patients with newly diagnosed CML-CP were stratified according to the Euro risk score and randomly assigned to dasatinib 100 mg/day or imatinib 400 mg/day. Confirmed CCyR by 12 months was the primary endpoint of the study and by 12 months was significantly higher for dasatinib (83 %, *P* < 0.001) than for imatinib (72 %), allowing also this drug to be approved as the first-line therapy by FDA and EMA. The best cumulative MMR rate by 12 months was also significantly higher for dasatinib (46 %, *P* < 0.0001) than for imatinib (28 %) [42]. Fewer progressions to accelerated phase or blast crisis (AP/BC) with dasatinib (1.9 %) than with imatinib (3.5 %) were already observed in the first report of these data [42]. After 5 years, molecular response rates continue to be higher for dasatinib compared with imatinib (rates of MMR 76 vs 64 %, *P* = 0.002 and rates of MR^4.5^ 42 vs 33 %, *P* = 0.025). Transformations to AP/BC on study or after discontinuation were lower with dasatinib (*n* = 12/259; 4.6 %) compared with imatinib (*n* = 19/260; 7.3 %). However, 5-year PFS and OS rates were similar across treatment arms (PFS 85 % dasatinib, 86 % imatinib; OS 91 % dasatinib, 90 % imatinib) [43]. A higher proportion of patients on dasatinib achieved BCR-ABL ≤10 % at 3 months (84 %) compared with those on imatinib (64 %). Patients who achieved BCR-ABL ≤10 % versus >10 % at 3 months showed improved PFS and OS and lower rates of transformation to AP/BP (PFS 89 vs 72 %, *P* = 0.0014; OS 94 vs 81 %, *P* = 0.0028; transformation *n* = 6/198 [3 %] vs *n* = 5/37 [14 %]) and imatinib (PFS 93 vs 72 %, *P* < 0.0001; OS 95 vs 81 %, *P* = 0.0003; transformation *n* = 5/154 3 % vs *n* = 13/85, 15 %) [24]. Concerning the AEs of dasatinib, the total incidence of pleural effusion after 5 years is 29 %, but most cases were grade 1 or 2 (67 out of 74) and discontinuation of dasatinib due to pleural effusion occurred in only 15 patients (6 % overall and 20 % of pts who experienced a pleural effusion). Arterial ischemic events were not common, occurring in 12 pts (5 %) on dasatinib and 6 pts (2 %) on imatinib [43]. More recently, however, one investigator-initiated study comparing dasatinib 100 mg OD vs imatinib 400 mg OD, although showing that the proportion of patients achieving CCyR was superior with dasatinib (84 % vs 69 %) as well as the 12-month molecular responses (MMR 53 vs. 35 %, *P* = 0.049; MR^4^ 25 vs. 10 %, *P* = 0.038), did not show any advantage in terms PFS as well as in terms of OS [44].

Finally, BELA is a phase 3 multicenter study comparing the efficacy and safety of bosutinib 500 mg OD with that of imatinib 400 mg OD [45]. In this study, CCyR by 12 months that was the primary endpoint of the study did not result to be significantly higher for bosutinib (70 %), compared with imatinib (68 %), and this did not allow bosutinib to be approved as the first-line therapy. These results have been jeopardized by the high rate of discontinuation mainly due to non hematologic drug-related AEs that occurred in the bosutinib arm (19 % rate of discontinuation in the bosutinib arm with respect to 5 % in the imatinib arm) and, in particular, the high rates of discontinuation due to diarrhea on bosutinib. However, MMR rates by 12 months were significantly higher for bosutinib (39 % bosutinib versus 26 % imatinib, *P* = 0.002) and there were numerically fewer progressions to AP/BC with bosutinib (2 %) than with imatinib (4 %) [45].

In conclusion, because of their higher inhibition capacity of the BCR-ABL TK, second-generation TKIs demonstrate some aspects of superiority compared to imatinib 400 mg OD as initial therapy for CML. This is revealed by a faster time to cytogenetic and molecular responses, with more patients achieving BCR-ABL ≤10 % at 3 months and by sustained higher cumulative responses, particularly by higher rates of very deep molecular responses like MR^4^ and MR^4.5^. The immediate clinical advantage of their use as front-line therapy could be represented by a lower rate of transformation, whereas on a longer run the advantage could be represented by a faster achievement of conditions allowing to reach and maintain a TFR state. However, 5-year OS are not statistically different with respect to imatinib and some observed long-term toxicity effects, like a higher rate of cardiovascular events, could raise concerns for their use, particularly in some categories of patients [46].

## High-dose imatinib for first-line treatment

Current treatment guidelines for CML recommend first-line therapy with imatinib at a dose of 400 mg/day. However this dosage may not be optimal for patients characterized by a genetic predisposition to a lower efficiency of the OCT-1 transporter, a pump regulating the intracellular influx and concentration of imatinib, who, on the contrary, could significantly benefit from higher initial imatinib dose [47]. Furthermore, phase 1 dose-finding trials demonstrated no dose-limiting toxicities at imatinib doses up to 1000 mg/day, and a dose–response relationship was observed and the best results with imatinib 400 mg were obtained when imatinib plasma concentration was at least 1000 μM/L [48]. This explains also why responses to imatinib are also so dependent on a perfect adherence to dosage and to scheduled treatment [49].

Based on these considerations, shortly after the approval of imatinib, a number of single-arm phase 2 studies were started to assess the efficacy and the safety of high-dose imatinib (800 mg) administration. These data compared favorably with historical controls [50–53]. In particular, the “Rationale and Insight for Gleevec High-Dose Therapy” (RIGHT) trial [52, 53], testing high-dose imatinib at 800 mg/day in previously untreated CML patients, showed a trend (although not statistically significant, *P* = 0.07), toward improved transformation free survival (TFS) with respect to historical controls treated with standard dose in the same institution [53]. An analysis of the kinetics of response in the RIGHT trial showed that 44 % of patients achieved a major MMR within 6 months of initiating therapy [52]. Compared with data from the IRIS trial [1], which showed a 6-month MMR rate of 21 % with standard-dose imatinib, these data suggest that high-dose imatinib can achieve more rapid responses. Another study, the TIDEL trial, used imatinib 600 mg/day to explore the concept of high-dose imatinib as initial therapy for CML in early CP [54]. When these data were compared with the imatinib arm of the IRIS trial, the CCyR rate was significantly improved with the higher dose (*P* < 0.001). However, the results obtained from ongoing randomized studies comparing first-line treatment with standard and high-dose imatinib are contrasting at the moment. The “Tyrosine kinase inhibitor OPtimization and Selectivity” trial (TOPS) is a phase III study involving 476 patients randomized in a 2:1 ratio to receive 800 or 400 mg/day imatinib [55]. In initial results, patients in the 800-mg arm achieved more rapid responses than the 400-mg arm at early time point months (3–6 months), although no significant difference was observed at 12 months (CCyR 70 vs 66 %, *P* = 0.35; MMR 46 vs 40 %, *P* = 0.20). A non significant trend was reported in patients with high Sokal scores for MMR rates at 12 months (41 vs 46 % for 800 vs 400 mg, *P* = 0.16). After 24 months of follow-up, no significant differences were reported in EFS, PFS, or OS. However, the lack of overall benefit with higher dose may be due in part to the frequent dose-reductions and treatment interruptions when starting with higher doses in this multicenter trial, as comparing patients in the high-dose imatinib arm with dose intensity (DI) ≥600 mg/day for the first 12 months vs dose intensity <600 mg/day, the results were statistically better for patients with DI ≥600 mg/day (CCyR rates at 12 months 89.6 vs 70.3 %, *P* < 0.000; MMR rates at 12 months 62.4 vs 34.1 %, *P* < 0.0001; MMR rates at 18 months 75.2 vs 40.3 %, *P* < 0.0001; time to MMR faster, *P* < 0.0001; duration of MMR longer, *P* = 0.0141). The ELN (Nordic countries, Italy, Turkey, Israel) study compared 400 versus 800 mg/day imatinib in 215 high-Sokal-risk patients, with a primary endpoint of CCyR at 12 months [56]. Although the results were not statistically significant, a clear trend toward higher rates of MMR with 800 mg/day compared with 400 mg/day was observed, MMR rate was 49 % in the high-dose arm compared with 41 % in the standard-dose arm [56]. The sample size selection, the fact that most of the patients treated with high-dose imatinib required a substantial dose decrease and, finally, the fact that the number of dropouts in the high-dose arm (18 %) was slightly higher than in the 400 mg arm could explain the lack of statistical significance in the high-risk patients in this trial [56].

Although these data do not apparently support the use of imatinib 800 mg as first-line treatment in newly diagnosed CP CML, more recently, in the Study IV trial of German CML Study Group, in which patients were randomized to receive imatinib 400 mg/day or 800 mg/day alone, or imatinib 400 mg/day in combination with interferon (IFN) alpha, a higher rate of MMR at 12 months was observed with tolerability-adapted imatinib 800 mg than with imatinib 400 mg (59 vs 44 %; *P* < 0.001). Median dose in the 800-mg arm was 628 mg/day, suggesting that treatment of early-phase CML with imatinib can be optimized and that early high-dose therapy followed by rapid adaptation to good tolerability can increase the rate of MMR at 12 months that in turn has been shown to be associated with improved survival [4].

Recently, these data have been confirmed by a randomized study comparing the rates of molecular, hematological, and cytogenetic response to IM400 vs. imatinib 400 mg twice daily (IM800) in which dose adjustments were allowed to maximize retention on study [57]. Molecular response (MR) at 12 months was deeper in the IM800 arm (4-log reduction of BCR-ABL1 mRNA 25 vs. 10 % of patients, *P* = 0.038; 3-log reduction 53 vs. 35 %, *P* = 0.049). Furthermore, in both arms, few patients relapsed, progressed, or died, but both PFS (*P* = 0.048) and RFS (relapse-free survival) (*P* = 0.031) were superior for IM800 [57].

## Combination therapy: imatinib plus interferon- alpha

Because of the established clinical benefit of IFN in CML treatment, combination therapy between this drug and imatinib always appeared appealing and it is under investigation in a number of clinical trials. In a phase 2 GIMEMA study of imatinib 400 mg/day plus PEG-IFNα2b 50–150 μg/week, CCyR and MMR rates were 70 and 47 % at 12 months, with a probability of maintaining CCyR at 5 years in responding patients of 94 % [58]. However, compliance to IFN was poor, with 87 % of patients discontinuing IFN within 2 years [58]. Some large randomized phase 3 trials are comparing imatinib monotherapy with combination treatment. In the open-label French SPIRIT trial, patients were randomized 1:1:1:1 to receive imatinib 400 mg/day, imatinib 600 mg/day, imatinib 400 mg/day plus cytarabine, or imatinib 400 mg/day plus pegylated interferon alpha (PEG–IFNα2a) [59]. A potential advantage for imatinib/IFN treatment was first observed in 18-month MMR (41 vs 52 vs 53 vs 62 %; *P* = 0.0001) and deep molecular response (4-log reduction of BCR-ABL transcripts, CMR4) (4 vs 7 vs 5 vs 15 %; *P* = 0.0013) rates and reconfirmed at later times [59]. However, further follow-up of SPIRIT is needed to establish whether these early differences confer a long-term survival advantage. Grade 3–4 neutropenia and/or thrombocytopenia during the first year was higher for combination arms (imatinib/cytarabine 41 %, imatinib/IFN 40 %) than in monotherapy arms (400 mg 8 %, 600 mg 14 %) [59]. Overall, 45 % of the patients discontinued IFN during the first 12 months. Interestingly, the duration of treatment with IFN had an impact on responses: in patients who have been treated for less than 4 months as compare to more than 12 months, rate of MMR, optimal molecular response MR4, and undetectable minimal residual disease increased from 48 to 82 %, 23 to 49 %, and 8 to 20 %, respectively [59]. A rather similar comparison has been performed within the German CML Study Group (Study IV), with an arm in which patients were receiving imatinib 400 mg/day in combination with unpegilated IFNα2beta [4]. With respect to imatinib 400 mg/day alone, 12-month CCyR rates were similar, 52 % for imatinib and 51 % for imatinib plus IFN, and 12-month MMR rates were 30 and 35 %, respectively [4]. After 5 years of follow-up, no difference was reported between arms in progression-free survival (PFS) or overall survival (OS) [4]. In a third trial performed by the Nordic CML study group, newly diagnosed chronic-phase CML patients with a low- or intermediate-Sokal-risk score and in imatinib-induced complete hematologic remission were randomized either to continue imatinib 400 mg/day or to receive a combination of pegylated IFN-α2b 50 μg weekly and imatinib 400 mg/day [60]. In the combination arm, 34 patients (61 %) discontinued PEGeg-IFN-α2b, most because of toxicity. The MMR rate at 12 months was significantly higher in the imatinib plus PEGeg-IFN-α2b arm (82 %) compared with the imatinib monotherapy arm (54 %; intention-to-treat, *P* = 0.002) and the MMR rate increased with the duration of PEGeg-IFN-α2b treatment (<12-week MMR rate 67 %, >12-week MMR rate 91 %) [60]. Finally, to determine whether adding PEGeg-IFN-α2b and GM-CSF to high-dose imatinib may further improve the cytogenetic and molecular response rates in CML patients, 94 patients were treated with imatinib 800 mg/day for the first 6 months and then randomized to continue high-dose imatinib alone or in combination with PEGeg-IFN-α2b at the dosage of 0.5 μg/kg per week and GM-CSF 125 mg/m^2^ three times weekly [61]. With a median follow-up of 54 months, no differences in the CCyR, MMR, and CMR rates were observed. However, the potential benefit of adding PEGeg-IFN-α2b and GM-CSF to imatinib may have been limited by the fact that, due to adverse events, all patients enrolled in the PEGeg-IFN-α2b arm discontinued this drug [61].

Reasons for these different findings between the French SPIRIT trial and the Nordic trial on one side and the German CML Study IV and the MDAnderson trial on the other side are not clear at the moment; however, multiple differences present in the protocols (i.e., the type of IFN used, patient populations, and trial designs) need to be considered.

In conclusion, although literature data are still rather controversial on the real efficacy of the association of imatinib plus IFN and higher rates of discontinuation are recorded due to IFN toxicity, the association of IFN and TKIs still appears particularly appealing for many investigators in view of a potential long-term effect on a higher rate of TFR. Indeed, a big study (CML V) has been recently initiated by the German CML Study Group to explore the safety and the efficacy of the association between nilotinib and IFNα2a.

## Conclusions

The choice of first-line treatment of CML in chronic phase is at the moment one of the hottest topics of debate among hematologists around the world. Imatinib has represented a fundamental step for the treatment of CML patients, totally changing their survival perspectives, and it has been able to save the lives of an incredible number of patients. Although changing from place to place, the cost of the drug is not low and this has certainly reduced the use of this drug in some low-income countries. The impact of this problem has been in part alleviated by the action of international charity programs supported by pharma companies like the GIPAP program by Novartis. As it already happened in some countries, the treatment with imatinib will certainly become more widely accessible when, after the imatinib patent expiration, the introduction of generic compounds will decrease the cost of the drug.

In spite of this, it should be recognized that the results that could be obtained with imatinib at the dosage of 400 mg/day are non optimal in approximately one third of the newly diagnosed CML patients and many investigational trials have been therefore started to try to further optimize first-line therapy [3]. For the moment, trials aiming to improve the outcome by increasing the imatinib dosage or by combining imatinib with IFN have provided in part contradictory results. However, the results obtained by the use of a tolerability-adapted imatinib dosage observed in the German CML study IV are very promising and have been recently confirmed by another independent study [57]. To be recommended, however, as standard first-line therapy for newly diagnosed CML patients, additional trials to clearly confirm this assumption are desired. On the other side, the use of the more potent second-generation TKIs dasatinib and nilotinib as initial treatment for CML, although not producing a significantly better OS with respect to standard-dose imatinib therapy, has been approved and registered by the FDA and EMA entities as potential alternatives to imatinib as first-line therapy for CML, mainly because of the faster and deeper responses induced by these drugs and for their capacity to prevent at least part the early progressions to AP/BC that may still occur during the first 2 to 3 years from diagnosis [40, 42]. This latter point, however, as mentioned, has not been confirmed for dasatinib in a second investigator initiated study and needs to be further explored [44].

The approval of nilotinib 300 mg/day and of dasatinib 100 mg/day in addition to imatinib as first-line therapy has introduced different therapeutic options for clinicians to treat newly diagnosed CML patients and, as specified also by the 2013 ELN guidelines where no preferential use of one of the three approved drugs is recommended, this gives to clinician the possibility to tailor the treatment according to the patients’ characteristics [12, 13]. Therefore, the use of the second-generation TKIs for all patients from the beginning or their use only for some subgroups of patients with high risk of progression or to initially start with imatinib 400 mg and then to switch to a second-generation TKI as soon as a non-optimal response is seen or only when an overt failure is recorded, this is at the moment mainly left to the choice of the doctor, who has of course to consider the balance between efficacy, toxicity, and affordable cost for each individual patient. Trials testing all possible therapeutic strategies are however presently ongoing and their results will certainly help clinicians to further make their decision.

At the moment, in the choice of initial CML therapy, we must consider also that the optimal endpoint to be pursued may vary from patient to patient. For an elderly patient, the attainment of an overall survival probability overlapping that of the corresponding control population without CML could be a sufficient target, but the expectations could be different for a younger patient who, aiming at a definitive cure, can also accept a more demanding therapeutic approach. This explains why CMR and the more precise definitions of molecular degrees of residual disease recently introduced (like MR^4^ and MR^4.5^) have become the primary endpoint of some clinical trials and also why, in the attempt to define parameters useful to identify patients with a higher probability of not relapsing after discontinuation, the number of studies with the final aim to increase CMR rates in view of possible therapy discontinuation is progressively increasing. As a fast initial response may be highly predictive of the patients’ final outcome, a more intense schedule for monitoring the response with cytogenetic and/or molecular analysis within the first semester of therapy is advisable even in common clinical practice, as clearly stated in the ELN and NCCN recommendations [12, 13].
